# A systematic comparison of machine learning algorithms to develop and validate prediction model to predict heart failure risk in middle-aged and elderly patients with periodontitis (NHANES 2009 to 2014)

**DOI:** 10.1097/MD.0000000000034878

**Published:** 2023-08-25

**Authors:** Yicheng Wang, Yuan Xiao, Yan Zhang

**Affiliations:** a Affiliated Fuzhou First Hospital of Fujian Medical University, Department of Cardiovascular Medicine, Fuzhou, Fujian, China; b Fujian Medical University, The Third Clinical Medical College, Fuzhou, Fujian, China; c Cardiovascular Disease Research Institute of Fuzhou City, Fuzhou, Fujian, China.

**Keywords:** heart failure, machine learning, national health and nutrition examination survey, NHANES, periodontitis, prediction model

## Abstract

Periodontitis is increasingly associated with heart failure, and the goal of this study was to develop and validate a prediction model based on machine learning algorithms for the risk of heart failure in middle-aged and elderly participants with periodontitis. We analyzed data from a total of 2876 participants with a history of periodontitis from the National Health and Nutrition Examination Survey (NHANES) 2009 to 2014, with a training set of 1980 subjects with periodontitis from the NHANES 2009 to 2012 and an external validation set of 896 subjects from the NHANES 2013 to 2014. The independent risk factors for heart failure were identified using univariate and multivariate logistic regression analysis. Machine learning algorithms such as logistic regression, k-nearest neighbor, support vector machine, random forest, gradient boosting machine, and multilayer perceptron were used on the training set to construct the models. The performance of the machine learning models was evaluated using 10-fold cross-validation on the training set and receiver operating characteristic curve (ROC) analysis in the validation set. Based on the results of univariate logistic regression and multivariate logistic regression, it was found that age, race, myocardial infarction, and diabetes mellitus status were independent predictors of the risk of heart failure in participants with periodontitis. Six machine learning models, including logistic regression, K-nearest neighbor, support vector machine, random forest, gradient boosting machine, and multilayer perceptron, were built on the training set, respectively. The area under the ROC for the 6 models was obtained using 10-fold cross-validation with values of 0 848, 0.936, 0.859, 0.889, 0.927, and 0.666, respectively. The areas under the ROC on the external validation set were 0.854, 0.949, 0.647, 0.933, 0.855, and 0.74, respectively. K-nearest neighbor model got the best prediction performance across all models. Out of 6 machine learning models, the K-nearest neighbor algorithm model performed the best. The prediction model offers early, individualized diagnosis and treatment plans and assists in identifying the risk of heart failure occurrence in middle-aged and elderly patients with periodontitis.

## 1. Introduction

Heart failure (HF) has a high probability of occurring in the end-stage of various cardiovascular diseases.^[1]^ The global prevalence of heart failure has been increasing in recent years.^[2]^ Heart failure is currently estimated to affect 6.5 million people in the United States, and by 2030, it is expected to affect more than 8 million people.^[3–5]^ Patients with heart failure often have a poor quality of life.^[6]^ Inflammation and fibrosis are believed to play a significant role in the remodeling of the heart that is typically linked with heart failure.^[7]^

More than 40% of individuals in the United States have periodontitis, a prevalent inflammatory oral disease that is the primary cause of tooth loss and is thought to influence the lives of individuals, their ability to chew, and nutritional status.^[8–13]^ Innate and adaptive immunity are both implicated in the pathophysiology of this chronic inflammatory illness of the oral cavity, which is characterized by the creation of periodontal pockets, loss of attachments, and resorption of alveolar bone.^[14]^ Periodontitis has grown to be a significant public health issue and a growing strain on healthcare systems as the world’s population ages.^[15,16]^

Periodontitis causes the development of cardiovascular inflammation and endothelial dysfunction, which is the principal mechanism through which periodontitis is linked to the development of cardiovascular disease.^[17]^ In patients with periodontitis, oral ecological dysregulation results in endotoxemia, which is also linked to an elevated risk of cardiometabolic abnormalities.^[18]^ Due to these causes, periodontitis has been linked for many years to the development of heart failure and other cardiovascular diseases.^[19,20]^ Some studies have shown that both periodontitis and heart failure are multifactorial. Factors such as smoking, diabetes, and advanced age are common in both diseases, and eliminating these factors can help progress in the treatment of both diseases.^[20,21]^Therefore, we included these factors in the covariates as well.

Machine Learning is an emerging field in medicine that integrates computer science and statistics into medical problems.^[22]^Machine learning has been applied to several areas of healthcare, including cardiology, cancer, and mental health.^[23–26]^Doctors and researchers can use machine learning algorithms to build prediction models that can predict the probability of a particular disease occurring.^[27–29]^ Machine learning can be broadly categorized into “supervised learning and “unsupervised learning depending on whether the model fitting is “supervised or “unsupervised”.^[30]^ When compared to other statistical methods, machine learning algorithms are better able to account for the interactions between variables, find hidden patterns, identify potentially significant predictor variables, find optimized algorithms between interesting outcomes and potential predictor variables by learning from dataset patterns, and perform more accurately evaluate clinical outcomes.^[31]^ Of course, it has also been asserted that the predictive power of logistic regression is comparable to that of machine learning methods.^[32–37]^ As it stands, there are few machine learning models for predicting the risk of heart failure in patients with periodontitis, and our study can help clinicians make treatment decisions by comparing machine learning algorithms that can build better predictive models that use clinical features to predict the risk of heart failure in patients with periodontitis.

## 2. Methods

### 2.1. Study population and data selection

**Figure 1.**
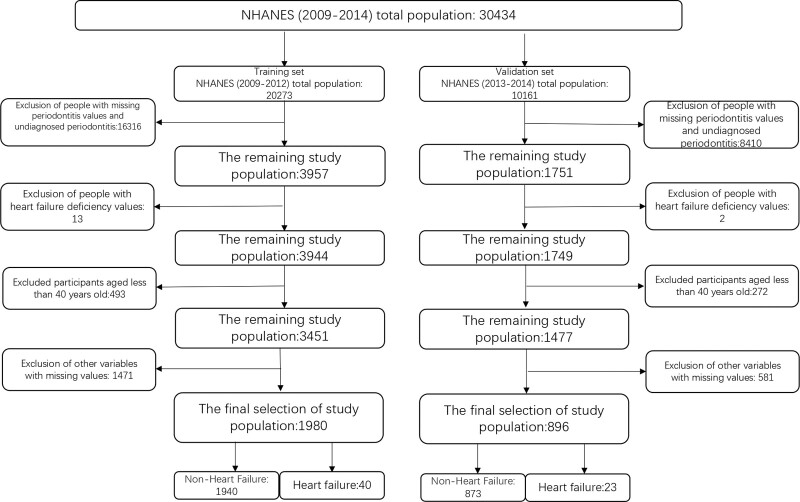
The flow chart of the study population for training set and validation set.

Data from the 2009 to 2014 National Health and Nutrition Examination Survey (NHANES), a research program created to evaluate the health and nutritional status of adults and children in the United States, were used in our study. The NHANES employs a number of intricate, stratified, multistage sampling designs to evaluate the health of Americans. The National Center for Health Statistics Research Ethics Review Committee authorized the survey protocol, and all participants completed informed consent forms. All procedures were conducted in accordance with relevant guidelines and regulations. Of the 30,434 subjects, we excluded those who were younger than 40 years of age and had missing values for smoking and drinking status, poverty-to-income ratio (PIR), diabetes mellitus (DM), coronary heart disease (CHD), myocardial infarction, hypertension, body mass index, education, marital status, race, sleep time on workdays, waist circumference, sedentary time, and physical activity. Two thousand eight hundred seventy-six participants aged 40 years and older who were diagnosed with periodontitis constituted our final study population. The full-mouth periodontal examination was performed by a dental hygienist who assessed the periodontal status of the participants. Participants aged 30 years and older were eligible for periodontal evaluation if they had at least 1 tooth (excluding the third molar) and did not meet any of the health exclusion criteria. Demographic characteristics can be disaggregated by gender (male, female), race (Mexican American, non-Hispanic white, non-Hispanic black, Hispanic, other race), marital status (unmarried, married or living with partner, married but currently living alone [separated, divorced, or widowed]), and education level (<9th grade, 9th to 11th grade, high school graduate, partial college or AA graduate or above) Classification. To calculate the PIR, household (or individual) income was divided by the survey year and state-specific poverty thresholds. Each participant’s smoking status was assessed by self-report and categorized into 3 groups based on their current smoking status: nonsmokers, ex-smokers who no longer smoke, and smokers. Never drinkers, former drinkers who now abstain from drinking, heavy drinkers (3 or more drinks per day for women and 4 or more for men), moderate drinkers (2 or fewer drinks per day for women and 3 or more for men), and light drinkers were the categories used to describe drinking status (not including above). Diabetes conditions can be categorized as: No, Prediabetes, Diabetes. Prediabetes, which includes impaired fasting glucose and impaired glucose tolerance, is a metabolic condition between diabetes and normoglycemia.^[38,39]^ According to the American Diabetes Association, the current definition of prediabetes in the United States includes a fasting blood glucose of 100 to 125 mg/dL, a post-load plasma glucose of 140 to 199 mg/dL, or an HbA1c of 5.7% to 6.4%.^[40]^ Physical activity, sleep time on workdays, sedentary time, hypertension, diabetes status, myocardial infarction status, and CHD were obtained by questionnaire. The flow chart of the study population screening is shown in Figure 1.

## 3. Ethical approval

The authors are accountable for all aspects of the work in ensuring that questions related to the accuracy or integrity of any part of the work are appropriately investigated and resolved. The study was conducted in accordance with the Declaration of Helsinki (as revised in 2013). All information from the NHANES program is available and free for public, so the agreement of the medical ethics committee board was not necessary. All the participants signed the written informed consent before participating in the study.

## 4. Constructing and validating predictive models

We also divided the study population in the validation set and training set into 2 groups based on the presence or absence of heart failure and compared baseline information. Our study identified independent risk factors for the risk of heart failure in subjects with periodontitis by weighted univariate and multivariate logistic regression. Statistically significant variables selected by stepwise regression were used as input variables. Variables with *P* < .05 in the univariate logistic regression analysis were included in the multivariate logistic regression analysis, and then the variables with *P* < .05 in the multivariate logistic regression analysis were selected as the final predictors to construct the machine learning model. In this study, a total of 1980 subjects with periodontitis from 2009 to 2012 were used as the training set and 10-fold cross-validation, and 896 subjects with periodontitis from 2013 to 2014 NHANES were used as the external svalidation set. We developed 6 ML algorithms such as logistic regression, K-nearest neighbor algorithm, support vector machine (SVM), random forest (RF), gradient boosting machine (GBM), and multilayer perceptron (MLP) to build the model on the training set. Receiver operating characteristic curve analysis is used in the external validation set to check the performance of each model. In both internal and external validation, the best performing model is defined based on the maximum areas under the receiver operating characteristic curve (AUC). The importance of the models was ranked according to the variables.

## 5. Statistical analysis

We employed NHANES sample weights in the baseline description and logistic regression analysis to achieve nationally representative values due to the complex sampling characteristics of NHANES. In the baseline information, continuous variables are expressed as means and standard errors. Categorical variables are expressed as frequencies and percentages. The *t* test was used to compare continuous variables between the 2 groups, and the chi-square test or Fisher exact test was used to determine the differences between groups when comparing categorical variables between the 2 groups. For participants with periodontitis, we utilized univariate and multivariate logistic regression to identify potential risk factors for heart failure. Odds ratio (OR) and 95% confidence intervals (CI) were utilized as effect estimates. *P* < .05 was considered statistically significant. All statistical analyses were performed using R software (version 4.3.0).

## 6. Results

### 6.1. Demographic characteristics

As the training set, we used a total of 1980 patients with periodontitis from the NHANES database from 2009 to 2012, and as the external validation set, we selected 896 periodontitis patients from the NHANES database from 2013 to 2014. The weighted baseline data of the training set are shown in Table 1. The mean age of the subjects was 57.24 years, and of these participants, 61.16% were male, 38.84% were female, 42.47% were non-Hispanic white, 23.89% were African American, 14.65% were Mexican American, 9.65% were Hispanic, and 9.34% were of other races. A total 1980 periodontitis participants were split into 2 groups based on whether or not they had heart failure. The differences between the heart failure group and the non-heart failure group were statistically significant (*P* < .05) in terms of age, PIR, physical activity, hypertension, CHD, myocardial infarction, and DM status. The baseline characteristics of the external validation set are similar to those of the training set. The mean age of the 896 subjects was 57.86 years. Of the 896 participants, 40.18% were non-Hispanic whites, 23.77% were African American, 15.51% were Mexican Americans, 8.82% were Hispanics, and 11.72% were Americans of other races. In comparison to those without heart failure, those with heart failure were older, had a smaller PIR, and engaged in physical activity less frequently. And there was statistical significance between the heart failure and non-heart failure groups in terms of age, CHD, and myocardial infarction (*P* < .05). The weighted detailed results of the general baseline information are shown in Table 2.

**Table 1:** Weighted baseline characteristics of training set.

Variables	Total	Non-heart failure	Heart failure	*P* value
N	1980	1940	40	
AGE (yr)	57.24 (0.34)	57.07 (0.36)	66.06 (1.20)	<.001
BMI (kg/m²)	28.42 (0.19)	28.37 (0.19)	31.47 (1.88)	.11
WC (cm)	99.81 (0.43)	99.65 (0.42)	108.68 (4.82)	.07
PIR	3.12 (0.06)	3.13 (0.06)	2.42 (0.33)	.04
Sleep (h)	6.80 (0.04)	6.80 (0.04)	6.60 (0.27)	.45
Sedentary (min)	334.05 (7.70)	333.39 (8.11)	370.15 (45.79)	.46
Exercise (min)	612.10 (21.55)	616.86 (21.80)	351.67 (84.04)	.003
Education				.001
<9^th^ grade	224 (11.31%)	218 (5.51%)	6 (8.77%)	
9–11^th^ grade	298 (15.05%)	293 (11.00%)	5 (15.10%)	
High school graduate	456 (23.03%)	445 (22.95%)	11 (19.56%)	
Some college graduate	535 (27.02%)	521 (30.54%)	14 (48.88%)	
College graduate or above	467 (23.59%)	463 (30.00%)	4 (7.69%)	
Marital				.86
Never married	177 (8.94%)	171 (8.40%)	6 (7.90%)	
Living with partner	1233 (62.27%)	1214 (65.37%)	19 (61.79%)	
Widowed/divorced	570 (28.79%)	555 (26.24%)	15 (30.31%)	
Race				.35
Non-Hispanic white	841 (42.47%)	825 (70.93%)	16 (56.88%)	
Non-Hispanic black	473 (23.89%)	457 (11.18%)	16 (21.42%)	
Mexican American	290 (14.65%)	286 (6.66%)	4 (6.24%)	
Other Hispanic	191 (9.65%)	188 (4.30%)	3 (3.01%)	
Other race	185 (9.34%)	184 (6.93%)	1 (12.45%)	
Sex				.52
Female	769 (38.84%)	753 (40.32%)	16 (34.22%)	
Male	1211 (61.16%)	1187 (59.68%)	24 (65.78%)	
Smoke				.18
Never	938 (47.37%)	922 (45.77%)	16 (26.67%)	
Former	600 (30.3%)	586 (31.17%)	14 (47.49%)	
Now	442 (22.32%)	432 (23.07%)	10 (25.83%)	
Alcohol				.38
Never	235 (11.87%)	227 (8.37%)	8 (15.72%)	
Former	409 (20.66%)	401 (17.91%)	8 (28.42%)	
Mild	726 (36.67%)	715 (40.06%)	11 (32.14%)	
Moderate	247 (12.47%)	241 (14.97%)	6 (9.23%)	
Heavy	363 (18.33%)	356 (18.70%)	7 (14.49%)	
MI				<.001
No	1909 (96.41%)	1886 (97.11%)	23 (68.09%)	
Yes	71 (3.59%)	54 (2.89%)	17 (31.91%)	
CHD				.43
No	1910 (96.46%)	1885 (97.23%)	25 (76.31%)	
Yes	70 (3.54%)	55 (2.77%)	15 (23.69%)	
Hypertension				<.001
No	685 (34.6%)	679 (39.29%)	6 (11.83%)	
Yes	1295 (65.4%)	1261 (60.71%)	34 (88.17%)	
DM				<.001
No	1337 (67.53%)	1321 (74.11%)	16 (27.23%)	
Prediabetes	179 (9.04%)	173 (8.58%)	6 (27.61%)	
Yes	464 (23.43%)	446 (17.31%)	18 (45.16%)	

BMI = body mass index, CHD = coronary heart disease, DM = diabetes mellitus, MI = myocardial infarction, PIR = poverty-to-income ratio, WC = waist circumference.

**Table 2:** Weighted baseline characteristics of validation set.

Variables	Total	Non-heart failure	Heart failure	*P* value
N	896	873	23	
AGE (yr)	57.86 (0.49)	57.53 (0.49)	67.97 (1.92)	<.001
BMI (kg/m²)	29.24 (0.32)	29.20 (0.31)	30.58 (1.37)	.29
WC (cm)	102.19 (0.83)	102.01 (0.80)	107.68 (3.03)	.07
PIR	2.79 (0.17)	2.79 (0.17)	2.72 (0.50)	.88
Sleep (h)	6.85 (0.08)	6.86 (0.08)	6.49 (0.46)	.44
Sedentary (min)	391.56 (7.59)	390.66 (7.70)	419.79 (41.13)	.5
Exercise (min)	593.35 (44.50)	599.81 (46.63)	391.20 (195.53)	.33
Education				<.001
<9^th^ grade	82 (9.15%)	81 (5.50%)	1 (2.85%)	
9–11^th^ grade	136 (15.18%)	128 (11.61%)	8 (31.03%)	
High school graduate	236 (26.34%)	233 (26.75%)	3 (23.47%)	
Some college graduate	259 (28.91%)	251 (29.91%)	8 (20.23%)	
College graduate or above	183 (20.42%)	180 (26.24%)	3 (22.42%)	
Marital				.86
Never married	86 (9.6%)	84 (10.26%)	2 (3.25%)	
Living with partner	537 (59.93%)	524 (59.89%)	13 (70.21%)	
Widowed/divorced	273 (30.47%)	265 (29.85%)	8 (26.54%)	
Race				.35
Non-Hispanic white	360 (40.18%)	345 (66.07%)	15 (86.30%)	
Non-Hispanic black	213 (23.77%)	210 (13.28%)	3 (6.77%)	
Mexican American	139 (15.51%)	137 (9.13%)	2 (3.60%)	
Other Hispanic	79 (8.82%)	77 (4.67%)	2 (1.94%)	
Other race	105 (11.72%)	104 (6.86%)	1 (1.39%)	
Sex				.46
Female	361 (40.29%)	349 (39.52%)	12 (50.04%)	
Male	535 (59.71%)	524 (60.48%)	11 (49.96%)	
Smoke				.58
Never	419 (46.76%)	410 (46.26%)	9 (30.66%)	
Former	265 (29.58%)	255 (30.13%)	10 (42.41%)	
Now	212 (23.66%)	208 (23.61%)	4 (26.93%)	
Alcohol				.24
Never	113 (12.61%)	109 (8.81%)	4 (12.95%)	
Former	197 (21.99%)	189 (19.10%)	8 (42.02%)	
Mild	314 (35.04%)	306 (35.40%)	8 (32.07%)	
Moderate	124 (13.84%)	122 (17.88%)	2 (4.91%)	
Heavy	148 (16.52%)	147 (18.80%)	1 (8.05%)	
MI				.001
No	852 (95.09%)	838 (96.99%)	14 (66.60%)	
Yes	44 (4.91%)	35 (3.01%)	9 (33.40%)	
CHD				<.001
No	855 (95.42%)	842 (95.32%)	13 (58.50%)	
Yes	41 (4.58%)	31 (4.68%)	10 (41.50%)	
Hypertension				.61
No	293 (32.7%)	290 (37.64%)	3 (27.95%)	
Yes	603 (67.3%)	583 (62.36%)	20 (72.05%)	
DM				.36
No	597 (66.63%)	588 (73.09%)	9 (59.23%)	
Prediabetes	83 (9.26%)	80 (8.14%)	3 (17.95%)	
Yes	216 (24.11%)	205 (18.77%)	11 (22.82%)	

BMI = body mass index, CHD = coronary heart disease, DM = diabetes mellitus, MI = myocardial infarction, PIR = poverty-to-income ratio, WC = waist circumference.

## 7. Univariate and multivariate logistic regression analysis

By using logistic regression analysis, our study identified independent risk variables for heart failure in individuals with periodontitis. The weighted results are displayed in Table 3. In a univariate analysis, the prevalence of heart failure in the population with periodontitis was significantly correlated with age, race, education, PIR, waist circumference, smoking status, hypertension, myocardial infarction, CHD, and DM status (*P* < .05). Age, race (Non-Hispanic Black, OR: 2.71, 95% CI:1.02–7.01, *P* = .046), the presence of myocardial infarction (OR: 7.17, 95% CI: 2.36–21.80, *P* = .002), and prediabetes (OR:5.86, 95% CI: 1.88–8.29, *P* = .005) were all statistically significant in a multivariable logistic regression analysis (*P* < .05).

**Table 3:** Weighted univariate and multivariate logistic regression analysis.

Variables	Univariate OR (95% CI)	*P* value	Multivariate OR (95% CI)	*P* value
Age (yr)	1.08 (1.05, 1.10)	<.001	1.08 (1.03, 1.14)	.003
BMI (kg/m²)	1.08 (1.00, 1.17)	.06	/	/
WC (cm)	1.04 (1.00, 1.08)	.05	/	/
PIR	0.76 (0.58, 0.99)	.04	/	/
Sleep (h)	0.89 (0.66, 1.21)	.44	/	/
Sedentary (min)	1.00 (1.00, 1.00)	.43	/	/
Exercise (min)	1.00 (1.00, 1.00)	.10	/	/
Education				
<9^th^ grade	Ref.	Ref.	Ref.	Ref.
9–11^th^ grade	0.86 (0.18, 4.04)	.85	/	/
High school graduate	0.54 (0.26, 1.10)	.09	/	/
Some college graduate	1.01 (0.32, 3.16)	.99	/	/
College graduate or above	0.16 (0.04, 0.73)	.02	/	/
Marital				
Never married	Ref.	Ref.	Ref.	Ref.
Living with partner	1.00 (0.29, 3.50)	.99	/	/
Widowed/divorced	1.23 (0.37, 4.02)	.73	/	/
Race				
Non-Hispanic White	Ref.	Ref.	Ref.	Ref.
Non-Hispanic Black	2.39 (1.02, 5.61)	.05	2.71 (1.02, 7.01)	.046
Mexican American	1.17 (0.44, 3.09)	.75	1.87 (0.52, 6.68)	.31
Other Hispanic	0.87 (0.26, 2.97)	.82	1.23 (0.28, 5.38)	.77
Other race	2.24 (0.30, 16.83)	.42	4.02 (0.67, 24.11)	.12
Sex				
Female	Ref.	Ref.	Ref.	Ref.
Male	1.30 (0.57, 2.97)	.52	/	/
Smoke				
Never	Ref.	Ref.	Ref.	Ref.
Former	2.61 (1.21, 5.64)	.02	/	/
Now	1.92 (0.53, 7.01)	.31	/	/
Alcohol				
Never	Ref.	Ref.	Ref.	Ref.
Former	0.84 (0.25, 2.84)	.78	/	/
Mild	0.43 (0.17, 1.06)	.07	/	/
Moderate	0.33 (0.10, 1.04)	.06	/	/
Heavy	0.41 (0.10, 1.66)	.20	/	/
MI				
No	Ref.	Ref.	Ref.	Ref.
Yes	15.77 (6.64, 37.45)	<.001	7.17 (2.36, 21.80)	.002
CHD				
No	Ref.	Ref.	Ref.	Ref.
Yes	10.88 (5.01, 23.62)	<.001	/	/
Hypertension				
No	Ref.	Ref.	Ref.	Ref.
Yes	4.82 (1.86, 12.52)	<.001	/	/
DM				
No	Ref.	Ref.	Ref.	Ref.
Prediabetes	8.76 (3.21, 23.92)	<.001	5.86 (1.88, 18.29)	.005
Yes	7.10 (2.45, 20.54)	<.001	2.99 (0.74, 12.14)	.12

BMI = body mass index, CHD = coronary heart disease, DM = diabetes mellitus, MI = myocardial infarction, OR = odds ratio, PIR = poverty-to-income ratio, WC = waist circumference.

## 8. The performance of machine learning models

**Figure 2.**
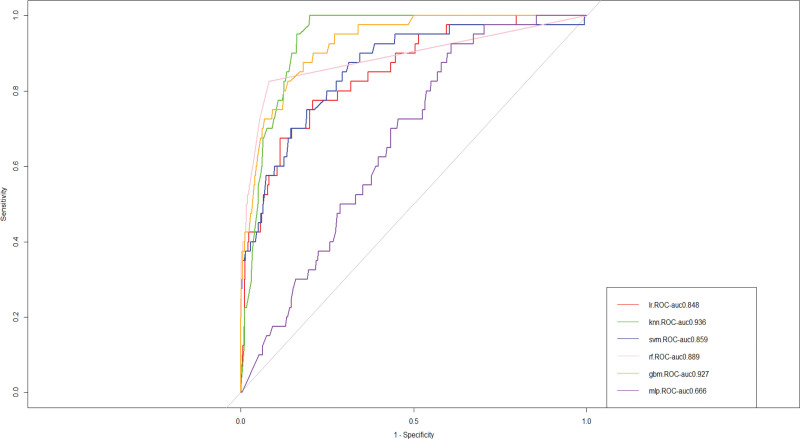
Area under the curve (AUC) values of 10-fold cross-validation. GBM = gradient boosting machine, KNN = K-nearest neighbor, LR = logistic regression, MLP = multilayer perception, RF = random forest, SVM = support vector machine.

**Figure 3.**
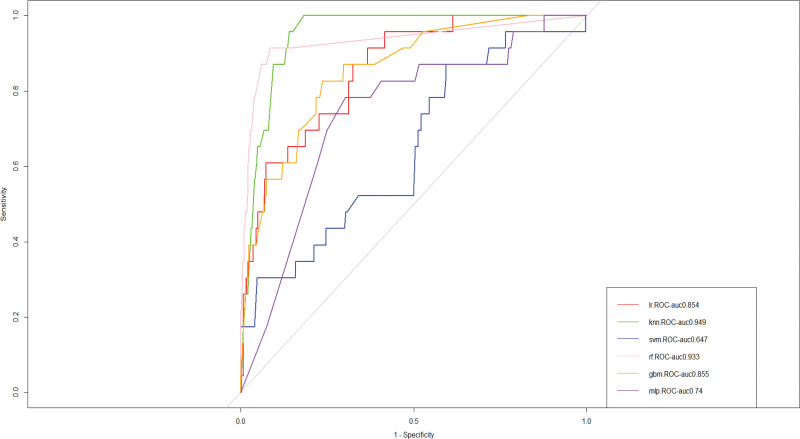
Area under the curve (AUC) values of external validation of machine learning algorithms. GBM = gradient boosting machine, KNN = K-nearest neighbor, LR = logistic regression, MLP = multilayer perception, RF = random forest, SVM = support vector machine.

We build prediction models in the training set using 6 machine learning algorithms: GBM, SVM, RF, K-nearest neighbor, logistic regression, and MLP. For internal validation, we evaluated the performance of the 6 machine learning models using 10-fold cross-validation, and the final k-nearest neighbor algorithm model outperformed the other 5 machine learning models in terms of predicting heart failure (AUC = 0.936), and the results are displayed in Figure 2. The k-nearest neighbor method model continued to outperform the other 5 machine learning algorithms in our external validation set (Fig. 3), with an AUC of 0.949. Consequently, as the final prediction model, we choose for the K-nearest neighbor algorithm model.

## 9. Relative importance of variables in machine learning algorithms

**Figure 4.**
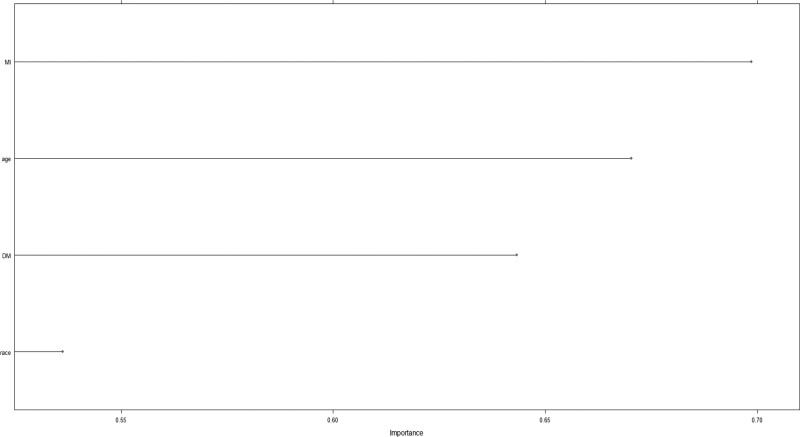
Relative importance ranking of each input variable for predicting models. DM = diabetes mellitus, MI = myocardial infarction.

The relative importance ranking of age, race, myocardial infarction, and diabetes in the model is shown in Figure 4. Among them, myocardial infarction status has the highest importance among the 4 variables, while race has the lowest importance.

## 10. Discussion

In this research, 896 periodontitis patients from the NHANES 2013 to 2014 were chosen for external validation, while 1980 periodontitis patients from the NHANES 2009 to 2012 were considered for model construction. We used 6 machine learning methods: logistic regression, K-nearest neighbor algorithm, SVM, RF, GBM, and MLP. In our study, the performance of 6 machine learning algorithms was assessed with regard to this, and finally, the k-nearest neighbor (KNN) model outperformed the others in terms of clinically predicting the risk of heart failure in participants with periodontitis. Age, race, myocardial infarction, and DM were significant independent risk variables for the probability of heart failure in participants with periodontitis, according to our multivariable logistic regression. The variables in the final model were ranked in descending order of importance as myocardial infarction, age, diabetes, and race.

Due to the fact that periodontitis is a common risk factor for heart failure, it is crucial to construct specialized treatment protocols and evaluate the significance of diagnostic accuracy in patients with periodontitis in order to lower the probability of developing heart failure.^[41–46]^ Our research demonstrates that myocardial infarction is a risk factor for heart failure in people with periodontitis. Heart failure is a manifestation of end-stage cardiovascular disease, including myocardial infarction.^[47,48]^ Studies have additionally demonstrated that periodontitis, a chronic condition that causes localized damage of the periodontal ligament and inflammatory bone loss brought on by oral microbes, is a risk factor for atherosclerosis and myocardial infarction.^[49,50]^ Further research is required to substantiate the outlook that myocardial infarction may increase the risk of heart failure development in people with periodontitis. According to the findings of numerous research, age is also a risk factor for heart failure. Younger participants in the Framingham Heart Study had a reduced absolute chance of developing heart failure than older participants, both with and without risk factors.^[51]^ Based on a Swedish study, the possibility of experiencing heart failure rises with age.^[52]^ The specific mechanism is probably, with increasing age, deterioration of heart structure and function during the aging process leads to an increased susceptibility to heart failure.^[53]^ In our analysis, DM was also found to be a risk factor for the development of heart failure. Many studies have also reported the effect of diabetes on heart failure.^[54–56]^ Research has revealed that cardiometabolic damage is directly related to how diabetes affects heart failure.^[57]^ Based on this, Halting the progression of diabetes is crucial for lowering the risk of heart failure. Even though it is regarded as the least important factor in our research, race is still a major predictor. Race has an impact on the prevalence of heart failure, and numerous studies have confirmed this.^[58–60]^ In our research, we discovered that black Americans were substantially more likely to experience heart failure than other racial groups. Consistent with our findings, a United States ARIC study of older persons without heart failure found that blacks had lower contractility than other racial groups and a higher risk of HFrEF.^[61]^ That according numerous studies, black males in their early and middle adulthood have worse systolic and diastolic function and are more prone to heart failure than other populations.^[61–63]^ Earlier epidemiological research has also suggested that young black persons have a 20-fold greater prevalence of heart failure than young white persons.^[64]^ More study is required to identify the explanatory processes underlying this racial disparity so that prospective interventions can be targeted because the specific mechanisms underlying this racial disparity are not currently fully known.

Also, even though earlier studies evaluated the risk of heart failure using machine learning models.^[65–67]^ However, the purpose of our study was to specifically use machine learning algorithms to predict the risk of heart failure in a population of patients with periodontitis. To our knowledge, this is the first predictive model to use machine learning to predict the risk of heart failure in patients with periodontitis. The significance of this study is that it is based on a risk assessment study of 2876 samples by comparing 6 machine learning algorithms, with the final KNN algorithm performed the best. Clinical treatment decisions can be guided by machine learning-based models that can assist doctors better anticipate the risk of heart failure in patients with periodontitis and implement the necessary measures.

There are some limitations to our study. First off, because this is a cross-sectional study, it was unable to determine the exact order of occurrences. Thus, further prospective studies are required to look at the causal connection between heart failure and periodontitis. Second, for model construction and evaluation, we employ a tenfold cross-validation approach. We use data from earlier years of the NHANES database for the external validation set, but we still need to confirm the final findings in other databases or with alternative cohorts. Third, while the NHANES database we utilize was built on the population of the United States, it still needs to be validated in other nations to see how the predictive model operates in different cultural settings.

## 11. Conclusion

In order to personalize the prediction of heart failure risk in people with periodontitis, our study created and evaluated 6 machine learning algorithms. We concluded that the KNN algorithm had the greatest model performance. The machine learning-based prediction models can aid physicians in making clinical decisions by assisting them in determining whether periodontitis patients are at risk for heart failure.

## Author contributions

**Formal analysis:** Yicheng Wang.

**Funding acquisition:** Yan Zhang.

**Methodology:** Yicheng Wang.

**Resources:** Yicheng Wang.

**Supervision:** Yan Zhang.

**Validation:** Yicheng Wang.

**Writing – original draft:** Yicheng Wang.

**Writing – review & editing:** Yicheng Wang, Yuan Xiao, Yan Zhang.
